# Is Japan at low risk for PFAS immunotoxicity?: human biomonitoring study in contaminated areas in Japan

**DOI:** 10.1265/ehpm.24-00047

**Published:** 2024-07-26

**Authors:** Zhaoqing Lyu, Kouji H. Harada, Junko Kimura-Kuroda, Yasuharu Tokuda

**Affiliations:** 1 Department of Health and Environmental Sciences, Kyoto University Graduate School of Medicine, Yoshida Konoe, Sakyo, Kyoto 606-8501, Japan; 2 NPO Japan Endocrine-disruptor Preventive Action; 3 Muribushi Okinawa Center for Teaching Hospitals

**Keywords:** Per- and polyfluoroalkyl substances, Serum, Human biomonitoring, Risk assessment, Okinawa

## Abstract

**Background:**

Per- and polyfluoroalkyl substances (PFAS) are now considered global contaminants posing health risks. Recent human biomonitoring data in Japan are presented.

**Methods:**

Human biomonitoring data from Japan, dating back to 2000, were reviewed. In addition, 399 serum samples collected in a primary care clinic in Urayasu City, Okinawa Island—one of the highest PFAS-exposed areas in Japan—between 2021 and 2022 were analyzed. Serum levels of four PFAS were compared with risk levels based on the assessment by Sonne et al. and the European Food Safety Agency.

**Results:**

The PFAS levels in the general population from various areas other than Hokkaido (16.1–43.5 ng/mL) are classified at moderate to severe risk for immunotoxicity based on the assessment.

**Conclusions:**

A portion of the Japanese population has had high exposure to PFAS and was at high risk of immunotoxicity, and this situation remained in PFAS-contaminated areas in the 2020s.

Per- and polyfluoroalkyl substances (PFAS) are now considered global contaminants posing health risks, including immunotoxic effects such as reduced efficacy of vaccine immunization [1, 2]. Recently, a global risk assessment for PFAS immunotoxicity, based on human biomonitoring data, was conducted [1]. In the study, Japan was classified in the low-risk category for immune deficiency (serum concentration of the sum of four PFAS (perfluorooctane sulfonate (PFOS), perfluorooctanoic acid (PFOA), perfluorohexane sulfonate (PFHxS) and perfluorononanoic acid (PFNA)), 0.7–9.5 ng/mL). However, this classification was based solely on data from the Hokkaido area, where PFAS exposure levels were low. Moreover, there is a concern regarding the literature survey for biomonitoring data in this report. The search terms were described as only “plasma, serum, PFAS, PFBS, PFHxS, PFHpS, PFOS, PFDS, PFOSA, PFHxA, PFHpA, PFOA, PFNA, PFDA, PFUnA, PFDoA, PFTrA, PFTeA and human”. In the search, PFAS were identified using abbreviated terms, which could miss literature where titles and abstracts used only spelled-out terms. Thus, there is concern that the study by Sonne et al. included a limited number of studies on human biomonitoring in Japan.

In this letter, we provide additional papers on human biomonitoring in Japan that were ignored in the study [1] (Table 1, [3–5]) as well as recently published data on pregnant women across Japan (Table 1, [6]). Additionally, we presented our recent survey results in drinking water-contaminated areas (Okinawa). Okinawa Island was reported to be one of the high PFOS and PFOA-exposed areas in Japan, with average PFAS levels of 14–44 ng/L detected in drinking water in 2013–2018 (https://www.eb.pref.okinawa.jp/userfiles/files/page/opeb/619/R06/2023_kako.pdf). The potential contamination source was suspected to be a leak of firefighting foam from Kadena U.S. military bases by the local government (https://www.eb.pref.okinawa.jp/sp/water/82/3017). The study was approved by the ethics committees at Muribushi Okinawa Center for Teaching Hospitals (14 June 2021, approval No. 2021-3) and Kyoto University (21 July 2021, approval No. R1478). Serum samples were collected from outpatients in a large primary care clinic in Urayasu City, Okinawa Island, from September 2021 to April 2022. Out of 403 outpatients with non-communicable diseases, 399 voluntarily participated (mean age, 53.4 yr; 219 males). Serum PFAS concentrations were determined by gas chromatography-mass spectrometry as previously reported [7]. As a result, although pregnant women in Japan had lower risk (6.23 ng/mL) [6], the PFAS levels in the general population from several areas, excluding Hokkaido, are classified at moderate to severe risk for immunotoxicity (16.1–43.5 ng/mL) based on the assessment by Sonne et al. and the European Food Safety Agency [1, 8].

**Table 1:** Serum 4 PFAS concentrations (ng/mL) in humans in Japan.

**Region**	**Participant**	**Exposure route**	**Value**	**Year**	**Gender**	**Age**	**Sample**	**PFHxS**	**PFOS**	**PFOA**	**PFNA**	**Σ4PFAS**	**Risk**	**Ref.**
Sapporo^a^	Mother-infant pairs	Numerous	Median	2002–2005	Women	Pregnant women with average of age around 30	514	–	5.7	1.2	–	6.9	low	[1]
Sapporo	Mother-infant pairs	Numerous	Mean	2002–2005	Women		428	–	5.6	1.4	–	7	low	[1]
Sapporo^b^	Mother-infant pairs	Numerous	Mean	2002–2005	Women		306	–	6	1.5	–	7.5	low	[1]
10 sites^c^	Men and women	Numerous	Median	2003–2004	Men/Women	38.9 ± 11.1/ 36.6 ± 10.3	200	–	18.3/11.7	4.6/4.4	–	22.9/16.1	high/moderate	[3]
Nagano	Women	Numerous	Median	2001–2005	Women	53.8 ± 10.5/ 54.0 ± 10.1 (case/control)	802	0.93	14.27	5.57	2.26	23.03	high	[4]
Western Tokyo^d^	Men and women	Waterborne	Mean	2020	Mix	50–80 (Mean: 68)	22	23.2	14.9	5.4	–	43.5	severe	[5]
15 regional centers	Mother-infant pairs	Numerous	Median	2011–2014	Women	31.0 [28.0, 35.0] (median [Q1, Q3])	25040	0.33	2.90	1.60	1.40	6.23	low	[6]
Okinawa^d^	Men and women	Waterborne	Mean	2021–2022	Mix	Mean: 53.4	399	7.1	7.6	2.8	2.1	19.6	high	

Value: Types of data for PFAS concentrations are presented. It should be noted that the mean level may be more overestimated than the median level, as the distribution of PFAS concentration in blood is generally right-skewed.

Risk: 1: low (0.7–9.5); 2: moderate (>9.5–17.5); 3: high (>17.5–31.9); 4: severe (>31.9) [1, 6].

Literature was selected from studies in 2000–2020 with participants ≥200 or studies conducted in contaminated sites [1].

^a^In the article [1], it was denoted as “Hokkaido”, but we confirmed that “Sapporo” (a city in Hokkaido) is more accurate.

^b^In the article [1], it was denoted as “National”, but we confirmed that “Sapporo” (a city in Hokkaido) is correct.

^c^The 10 sampling sites included Akita City, Sendai City, Takayama City, Matsuoka Town of Fukui prefecture, Kyoto City, Osaka City, Nishinomiya City, Shimonoseki City, Kochi City and Naha City [2].

^d^Survey in Western Tokyo was conducted by NPO Japan Endocrine-disruptor Preventive Action (Kimura-Kuroda), and in Okinawa by Tokuda.

As shown here, a portion of the Japanese population has had high exposure to PFAS and was at significant risk of immunotoxicity. This situation persisted in PFAS-contaminated areas into the 2020s. Identification of sources of contamination and reduction in exposure to PFAS should be warranted to prevent future health risks.

## Abbreviations

EFSA: European Food Safety Agency
PFAS: Per- and polyfluoroalkyl substances
PFOS: perfluorooctane sulfonate
PFOA: perfluorooctanoic acid
PFHxS: perfluorohexane sulfonate
PFNA: perfluorononanoic acid

## Declarations

### Animal welfare

N/A.

### Data availability statement

The data that support the findings of this study are available from the corresponding author upon reasonable request.
